# Cortisol in mother’s milk across lactation reflects maternal life history and predicts infant temperament

**DOI:** 10.1093/beheco/aru186

**Published:** 2014-10-31

**Authors:** Katie Hinde, Amy L. Skibiel, Alison B. Foster, Laura Del Rosso, Sally P. Mendoza, John P. Capitanio

**Affiliations:** ^a^Department of Human Evolutionary Biology, Harvard University, 11 Divinity Avenue, Cambridge, MA 02138, USA,; ^b^Brain, Mind, and Behavior Unit, California National Primate Research Center, University of Califoria, One Shields Avenue, Davis CA 95616, USA,; ^c^Nutrition Laboratory, Smithsonian National Zoological Park, 3001 Connecticut Avenue NW, Washington, DC 20008, USA,; ^d^Department of Biological Sciences, Auburn University, 101 Rouse Life Science Rd, Auburn, AL, 36849, USA,; ^e^Division of Early Childhood, Mills College, 5000 MacArthur Blvd, Oakland, CA, 94613, USA, and; ^f^Department of Psychology, University of California Davis, One Shields Ave, Davis, CA, 95616, USA

**Keywords:** behavioral phenotype, breast milk composition, developmental programming, glucocorticoids, life-history theory, personality.

## Abstract

The maternal environment exerts important influences on offspring mass/growth, metabolism, reproduction, neurobiology, immune function, and behavior among birds, insects, reptiles, fish, and mammals. For mammals, mother’s milk is an important physiological pathway for nutrient transfer and glucocorticoid signaling that potentially influences offspring growth and behavioral phenotype. Glucocorticoids in mother’s milk have been associated with offspring behavioral phenotype in several mammals, but studies have been handicapped by not simultaneously evaluating milk energy density and yield. This is problematic as milk glucocorticoids and nutrients likely have simultaneous effects on offspring phenotype. We investigated mother’s milk and infant temperament and growth in a cohort of rhesus macaque (*Macaca mulatta*) mother–infant dyads at the California National Primate Research Center (*N* = 108). Glucocorticoids in mother’s milk, independent of available milk energy, predicted a more Nervous, less Confident temperament in both sons and daughters. We additionally found sex differences in the windows of sensitivity and the magnitude of sensitivity to maternal-origin glucocorticoids. Lower parity mothers produced milk with higher cortisol concentrations. Lastly, higher cortisol concentrations in milk were associated with greater infant weight gain across time. Taken together, these results suggest that mothers with fewer somatic resources, even in captivity, may be “programming” through cortisol signaling, behaviorally cautious offspring that prioritize growth. Glucocorticoids ingested through milk may importantly contribute to the assimilation of available milk energy, development of temperament, and orchestrate, in part, the allocation of maternal milk energy between growth and behavioral phenotype.

## Lay Summary

In monkeys, high cortisol and changes in cortisol levels in mother’s milk are associated with more nervous and less confident infants. Sons are more sensitive than are daughters to changes in cortisol in mother’s milk across lactation. Females that are earlier in their reproductive career tend to have higher cortisol in their milk. Mothers may be “programming” behaviorally cautious offspring that prioritize growth through cortisol signaling.

## INTRODUCTION

Since the early 20th century, scientists have revealed a multitude of maternal effects on offspring phenotype. Among plants, birds, insects, reptiles, fish, and mammals, the maternal ecology that offspring experience reflects maternal characteristics, life-history trade-offs, and ecological conditions. This maternally mediated ecology of development has been demonstrated to influence offspring mass/growth, metabolism, reproduction, neurobiology, immune function, and behavior (Smith 1919; Moore et al. 1927; Mousseau and Fox 1998; Räsänen and Kruuk 2007; Champagne 2008; Groothuis and Schwabl 2008; Skibiel et al. 2009; Ezard et al. 2014; Mateo 2014). Scholars posit that these reaction norms in offspring phenotype variably reflect deficits from early insults (Suomi 1997; Sameroff and Rosenblum 2006; Monaghan 2008), adaptive calibration to maternal resources (Wells 2007, 2010, 2014; Love and Williams 2008), or predictive adaptation to adult environment (Barker 2002; Gluckman et al. 2005; Monaghan 2008). Investigations of early life “programming” of offspring phenotype among mammals have largely addressed physiological influences at the maternal–placental–fetal interface, particularly nutritional transfer and hormonal signaling (Benyshek 2013; Rutherford 2013). Reduced nutrient transfer during fetal development causes intrauterine growth restriction as evidenced by small-for-gestational-age neonates (Cetin and Alvino 2009; Rutherford 2013). Similarly, prenatal exposure to elevated maternal glucocorticoids, the end product of the hypothalamic-pituitary-adrenal axis (corticosterone in rodents, cortisol in primates), causes low birth weight and compromised neurodevelopment and immune function (Coe and Lubach 2000; Seckl 2004; Murphy et al. 2006; Singh et al. 2012).

Among mammals, substantial physiological investment continues into the postnatal period. Lactation imposes significant energetic costs because mothers synthesize milk not only to support continued offspring growth and development (Gittleman and Thompson 1988; Prentice and Prentice 1988; Emery Thompson 2013) but also to support offspring behavioral activity (Hinde and Capitanio 2010; Hinde 2013). Mother’s milk, therefore, is an important physiological pathway for nutrient transfer and glucocorticoid signaling that potentially influences offspring growth and behavioral phenotype (Peaker and Neville 1991; Catalani et al. 2011; Hinde 2013; Dettmer et al. 2014).

Mother’s milk has been associated with offspring behavioral phenotype in a handful of taxa (reviewed in Hinde 2013; Dettmer et al. 2014). Collectively, these studies indicate that maternal-origin glucocorticoids ingested via milk are implicated in the development of behavioral phenotype, but the directionality and the magnitude of these effects can be different between sons and daughters. A series of systematic experimental manipulations in laboratory rats (*Rattus norvegicus*) over several decades demonstrated that exposure to elevated glucocorticoids through mother’s milk increased exploratory behavior, reduced anxiety, and enhanced learning in juvenility and adulthood (Angelucci et al. 1983, 1985; Catalani et al. 1993, 2000, 2002; Casolini et al. 1997; Meerlo et al. 2001; reviewed in Macrì et al. 2011; Catalani et al. 2011). Studies in monkeys and humans have measured “unmanipulated” cortisol concentrations in mother’s milk contemporaneously with infant temperament assessments. Cortisol concentrations in the milk at 3 months postpartum were associated with more Confident temperament (confident, bold, active, curious, and playful) in sons (Sullivan et al. 2011) but greater “Negative Affectivity” (infant’s tendency toward fear, sadness, discomfort, anger/frustration, and reduced soothability) in daughters (Grey et al. 2013).

Studies of the effects of glucocorticoids in mother’s milk, however, have been handicapped by not simultaneously evaluating milk energy density and yield (Peaker and Neville 1991; Hinde 2013). Indeed the concentration of glucocorticoids in milk will be correlated with milk composition and yield (*Bos taurus*, Fukasawa et al. 2008; *Macaca mulatta*, Sullivan et al. 2011) because of the functional and biophysical properties of glucocorticoids within the mammary gland. For example, glucocorticoids are lipophilic molecules and contribute to nutrient flux and protein synthesis and prevent cell death in the mammary gland (Motil et al. 1994; Akers 2002; Berg et al. 2002). This poses a particular problem because the enhanced behavioral and learning outcomes of glucocorticoids may be an artifact of, or partially explained by, increased nutrient transfer in milk. Previously, we demonstrated that available milk energy (AME)—the product of milk yield and milk energy density from fat, protein, and sugar concentrations—during early behavioral development was predictive of infant Confident temperament months later in captive *M. mulatta* (Hinde and Capitanio 2010), similar to the effects of cortisol concentrations in milk (Sullivan et al. 2011). The behavioral trait adjectives of confident, bold, active, curious, and playful that together manifest as the factor “Confident,” are a priori expected to be sensitive to energy balance. That sensitivity may be in part mediated by glucocorticoids.

Glucocorticoids not only regulate the “stress response” but are instrumental in the routine metabolism of carbohydrates, proteins, and fats (Sapolsky et al. 2000; Djurhuus et al. 2004; Barrett et al. 2010; Nyberg 2013). Individual differences in metabolism and behavioral phenotype have been posited to be correlated (Careau et al. 2008; Biro and Stamps 2010; Houston 2010), and metabolism has been associated with behavioral phenotype in adults (*Mus domesticus*, Rezende et al. 2009; *Peromyscus maniculatus*, Careau et al. 2011; *Canis familiaris*, Careau et al. 2010; *Microtus oeconomus*, Lantová et al. 2011). The substantial literature on the early life canalization of metabolism suggests the possibility that behavioral phenotype and metabolism are co-organized to some extent by mother’s milk (Peaker and Neville 1991). Glucocorticoids ingested through milk may, therefore, importantly contribute to the assimilation of AME, development of temperament, and orchestrate, in part, the allocation of maternal milk energy between growth and behavioral phenotype (Hinde 2013).

Although the concentration of glucocorticoids in milk shows substantial variation, the sources of that variation across individuals and across time within individual remains unclear. In the rodent studies to date, glucocorticoids have been experimentally elevated without assessing baseline or naturally occurring concentrations of glucocorticoids in milk. Maternal characteristics associated with glucocorticoid concentrations in human and monkey milk have not been reported (Sullivan et al. 2011; Grey et al. 2013). Maternal plasma concentrations of glucocorticoids predict about 35% of the variance of concentrations in milk (Sullivan et al. 2011). As such, glucocorticoids in mother’s milk may reflect maternal condition and ecology, already known to be associated with milk energy density and yield (*C. elaphus hispanicus*, Landete-Castillejos et al. 2010; *B. taurus*; Wathes et al. 2007; *Sigmodon hispidus*, Rogowitz 1998; humans and nonhuman primates reviewed in Hinde and Milligan 2011). Interestingly, among American red squirrels (*Tamiasciurus hudsonicus*), perceptions of population density in the maternal environment increases maternal circulating glucocorticoids, accelerating offspring growth (Dantzer et al. 2013). The accumulated studies, taken together, further substantiate the hypothesis that glucocorticoids ingested via milk shape behavioral phenotype of young as a function of maternal and infant life-history trade-offs.

Here, we present the first study in any mammal to evaluate, simultaneously and longitudinally, natural variation in mother’s milk cortisol and AME in relation to maternal and infant characteristics. We predicted that cortisol concentrations in milk, controlling for AME, would be associated with infant temperament along dimensions of confidence and nervousness. Due to the previously reported sex-differentiated effects of glucocorticoids in milk in this colony specifically (Sullivan et al. 2011), we predicted that these effects would be greater in sons than in daughters. Previous studies have evaluated glucocorticoids in mother’s milk nondynamically at a single time point or after artificial elevation by an unknown amount above baseline. However, hormones exert physiological effects dynamically and critical windows of sensitivity are likely to differ between sons and daughters (Menger et al. 2010; Brummelte et al. 2012). We, therefore, considered changes in glucocorticoid concentrations and available energy in milk across lactation, in addition to absolute values at early and peak lactation, under the prediction that changes in milk across time may be physiologically salient to offspring. Although glucocorticoid exposure during very early development is generally associated with poor growth, we predicted that cortisol concentrations in milk would be positively associated with infant growth, as increased maternal glucocorticoids have been implicated in accelerated offspring growth rates (Dantzer et al. 2013).

To test our predictions, we studied animals living in the outdoor field corrals at the California National Primate Research Center (CNPRC) and that had undergone a standardized colony-wide BioBehavioral Assessment (BBA; Capitanio et al. 2005; Golub et al. 2009; Hinde and Capitanio 2010). Rhesus monkeys have been an ongoing and valuable animal model of maternal–infant dynamics (Hinde and White 1974; Berman 1980a; Machado 2013) and their behavior, temperament, and physiology are well described (Freeman and Gosling 2010; O’Sullivan et al. 2013; Phillips et al. 2014). Moreover, in our outdoor colony, mother–infant dyads are able to engage in the full suite of species-typical behavior and infants develop in the context of a social group similar in composition to wild-living rhesus macaques (Hinde and Capitanio 2010). Milk was obtained at 2 time points associated with important behavioral development and milk synthesis milestones (Hinde et al. 2009; Machado 2013). We assessed whether measures of temperament were better predicted 1) by AME and cortisol concentrations when infants first begin behaviorally exploring beyond their mother at 1 month of age, a critical window of neurodevelopment (reviewed in Hinde and Capitanio 2010; Machado 2013) versus 2) at peak lactation (Hinde et al. 2009), approximately 3.5 months of age, when behavioral assessment was conducted, or 3) by dynamic changes between the 2 time points.

## METHODS

### Subjects

Mother–infant dyads (*N* = 108) were recruited from 13 different social groups in the outdoor breeding colony at the CNPRC during infant birth seasons in 2006 (*N* = 42) and 2010 (*N* = 66). Each social group is composed of close kin, distantly related kin, and nonkin in a social structure similar to that found among wild-living rhesus groups. Subjects had produced 1–18 infants in their reproductive career at the time of the study; however, 34% were primiparous (37/108). Just over half of the mothers were rearing daughters (61/108, 56%). Subjects were fed a commercial diet twice-daily (Purina Monkey chow) supplemented with fresh produce semiweekly. Subjects were housed in 0.2-ha corrals that included multiple structures for climbing and several food distribution stations. Linear hierarchies generated by the behavioral management division at the CNPRC, based on standardized observations of social interactions, were used to categorize social rank of mothers. We split the linear hierarchies into thirds to assign “high,” “middle,” and “low” rank to individual mothers, with the exception that study mothers were assigned the rank category that characterized the majority of their matriline if the arbitrary tertile assignment recategorized them from their matrilineal kin (Hinde et al. 2009; Hinde and Capitanio 2010).

### Milk collection

Milk was collected using standardized methods from mothers (Hinde et al. 2009; Hinde and Capitanio 2010) at 1-month postpartum and again between 3 and 4 months postpartum during peak lactation (Oftedal 1984; Riek 2008). In the early postnatal period, at 1 month of infant age (mean ± standard deviation [SD] = 34±2.5 days), infants and mothers were captured in their outdoor enclosures between 7:30 and 9:00 AM and were relocated together to temporary housing. To prevent nursing, mothers were placed in mesh jackets and allowed a standardized period of milk accumulation for 3.5–4h. This allowed infants to remain in contact with their mother during this period. Between 11:30 and 13:00, mothers were sedated with ketamine hydrochloride (5–10mg/kg intramuscularly) and administered a nonphysiological dose of exogenous oxytocin [2 IU/kg (0.1mL/kg) intramuscularly] to stimulate milk letdown. Milk was collected by hand, and mammary glands were fully evacuated to prevent sampling bias (Oftedal 1984). Following collection, milk samples were placed directly into wet ice and transported from the procedure room to the wet lab where they were vortexed for 5 s, aliquoted into cryovials, and frozen at −80 °C. Milk collection procedures used in this study have been described in further detail elsewhere (Hinde et al. 2009). At the 1-month time point, after the mother had recovered from sedation, mothers and infants were returned to their social group the same day. During peak lactation, between 3 and 4 months of infant age (mean ± SD = 109±10 days), infants and mothers were again captured between 7:30 and 9:00 AM and separated, and infants were relocated to a novel environment for 25h, during which infants’ behavioral responsiveness and temperament were assessed (Capitanio et al. 2005, 2006; Hinde and Capitanio 2010; see below). Following 3.5–4h of milk accumulation, milk was again collected and handled using identical techniques as described for the earlier time point.

### Milk cortisol

Cortisol concentrations in milk were measured using a modified radioimmunoassay for salivary samples previously validated for rhesus macaque milk (Sullivan et al. 2011). Once thawed, 200 µL of sample was diluted with 600 µL of distilled water (1:4 dilution) and centrifuged at 3000rpm for 10min (Sorvall ST40R). Centrifugation resulted in the separation of the aqueous component from suspended particles in milk and a floating lipid layer. Removal of lipids and suspended particles through centrifugation resulted in reduced cortisol concentrations that were 81% of whole milk values (Sullivan et al. 2011). Concentrations of cortisol in the aqueous component were estimated in duplicate using commercial coated tube RIA kits (Siemens Medical Solutions Diagnostics, Los Angeles, CA). Assay standards were diluted to concentrations ranging from 2.76 to 317.4 nmol/L; the modified assay displayed a linearity of 0.98 and a least detectable dose of 1.3854 nmol/L. Diluted samples and label were incubated at room temperature for 3h before gamma counter reading. Pooled samples were included for quality control purposes: the interassay coefficient of variation (CV) was 5.51% and intra-assay CV was 2.97%. The average ± SD intrasample CV (duplicate determinations of individual samples) was 2.03±1.7%.

### Available milk energy

An index of AME was calculated as the product of milk energy density (kcal/g) and milk yield (g) for each mother at the 1- and 3.5-month time points (Hinde 2009; Hinde and Capitanio 2010). This index of AME represents relative differences in milk synthesis among mothers under controlled experimental conditions. Proximate analyses of milk composition (concentrations of fat, protein, and sugar) from the 2006 study season (*N* = 42) were conducted at the Nutrition Laboratory, Smithsonian National Zoological Park, Washington, DC, and reported previously (Hinde et al. 2009). We measured fat concentration via a modified Rose-Gottlieb method, calculated protein based on crude nitrogen content, and sugar concentration from the phenol–sulfuric acid method described in precise detail elsewhere (Hinde et al. 2009; Hood et al. 2009). Remaining aliquots of the 2006 samples were used to calibrate a MIRIS milk analyzer (Miller et al. 2013) for macaque milk samples in the Comparative Lactation Laboratory, Harvard University. Following calibration, samples collected in the 2010 study season (*N* = 66) were assayed in duplicate on the MIRIS milk analyzer in concert with 610 additional samples for other ongoing studies. The mean ± SD CV of duplicate determinations in macaque milk for fat, protein, and sugar were 1.0±1.0%, 1.5±2.0%, and 1.0±1.0%, respectively. Pooled bovine whole milk samples were used as controls at the beginning and end of each assay run. Based on these bovine samples, the interassay CV for fat was 0.52%, for protein was 0.72%, and for sugar was 0.36%, and the intra-assay CV for fat was 1.65%, for protein was 1.24%, and for sugar was 1.38%. The gross energy of milk, also referred to as energy density, was calculated as 9.11 kcal/g for fat, 3.95 kcal/g for sugars, and 5.86 kcal/g for protein, which allowed for an estimate of the total energy density of milk (kcal/g) (Oftedal 1984; Hinde and Milligan 2011). Milk yield was measured gravimetrically as total sample (g) obtained by full evacuation of milk from both mammary glands after a standard period of milk accumulation (3.5–4h). This method of estimating relative differences in milk production among individuals has been used for other primates (Ota et al. 1991), validated with doubly labeled water (Tardif et al. 2001), and has been associated with infant mass and growth in the CNPRC population (Hinde 2009; Hinde et al. 2009).

### BBA and temperament

All 108 infant subjects were part of an ongoing BBA program at the CNPRC described in detail elsewhere (Capitanio et al. 2005, 2006; Golub et al. 2009). Briefly, cohorts of up to 8 animals at a time were relocated to an indoor testing area when infants were 3–4 months of age, and each animal in a cohort was housed in an individual holding cage (60cm × 65cm × 79cm, Lab Products, Inc., Maywood, NJ), containing a cloth diaper, a stuffed terrycloth duck, and a novel, manipulable object. Over the next 25-h period, behavioral data were collected in a variety of standardized situations (described in Capitanio et al. 2006; Golub et al. 2009); at the conclusion of this period, infants were returned to their mothers, and mother–infant pairs were returned to their outdoor enclosures. The present study relied on measures of infant temperament that were obtained using subjective observer ratings (Weinstein et al. 2008), which reflected an overall “thumbnail” portrait of the animal’s functioning during the entire BBA: at the end of the 25-h period, an observer rated each infant using a list of 16 trait adjectives (Golub et al. 2009, Table 3) and a 7-point Likert scale for each trait. Assessment of inter-rater agreement and reliability for the data collection has been previously published (Weinstein and Capitanio 2008), and Golub et al. (2009) report details of the exploratory and confirmatory factor analyses that were performed on data from 1284 infants. These analyses revealed a 4-factor structure, each named for the adjective with the highest factor loading: “Vigilant” (vigilant, NOT depressed, NOT tense, NOT timid), “Gentle” (gentle, calm, flexible, curious), “Confident” (confident, bold, active, curious, playful), and “Nervous” (nervous, fearful, timid, NOT calm, NOT confident). The traits preceded by the word “not” reflect a negative loading in the factor analysis. Scales were constructed and *z*-scored, as previously described (Golub et al. 2009). Cronbach’s alpha for each dimension ranged from 0.6 to 0.9.

### Statistical analysis

Descriptive statistics were calculated for cortisol in milk and are reported as raw values (nmol/L). Cortisol concentrations at early and peak lactation were not normally distributed after evaluation with a Shapiro–Wilk goodness-of-fit test and were subsequently log-transformed for statistical analysis. Milk parameters (cortisol and AME) at early and peak lactation did not differ between the 2006 and 2010 sampling seasons and so the 2 seasons were combined in analyses. Maternal age and parity were highly correlated (*r* ^2^ = 0.94, *P* < 0.0001). To avoid violation of collinearity during model construction, parity was used instead of age because of its greater biological relevance: behavioral care and physiological investment improve as a function of reproductive experience (Fairbanks 1996; Cameron et al. 2000; Hinde et al. 2009; Machado 2013). The association between cortisol and milk fat and yield was evaluated using a multiple regression model in which time point (early vs. peak lactation) was nested within maternal ID as random effects to account for the repeated measures. To test outcome measures of cortisol in milk at each time point, infant temperament, and infant growth across time, multiple regression models were constructed using Akaike information criterion corrected for finite sample sizes (AICc) to retain the fewest parameters that appreciably explained variation in outcome measures (Akaike 1974; Burnham and Anderson 2004). Exploratory analysis of temperament factors revealed high correlations among the 4 temperament factors. Only “Confident” and “Nervous” temperament factor *z*-scores are considered here because they reflected behavioral phenotypes previously associated with cortisol in milk; they also had the lowest correlation with one another among the temperament factors (*r* = −0.5). Models with infant temperament factor as the outcome variable were constructed independently for sons and daughters as sex differences in sensitivity to milk cortisol and critical windows of sensitivity were predicted. When the sample was divided by infant sex, maternal parity was dichotomized into “primiparous” and “multiparous” categories. Statistical analyses were performed using JMP Pro 10 (SAS Institute, Inc.) with the exception that calculation of the intraclass correlation on cortisol concentrations at early and peak lactation was conducted in R package (Wolak et al. 2012; R Core Team 2014). Significance was accepted at *P* ≤ 0.05.

## RESULTS

### Cortisol in milk

**Figure 1:**
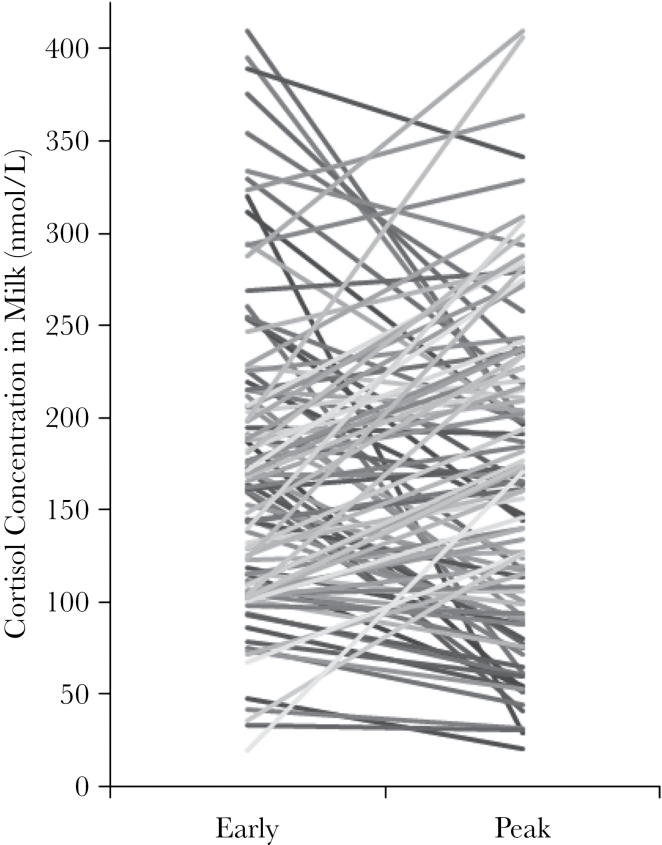
Concentration of cortisol in milk (nmol/L) for each individual rhesus macaque mother at early and peak lactation (1 month and 3–4 months, respectively). Each line represents an individual female (*N* = 107 shown, *y*-axis truncated at 425 nmol/L excludes one subject) and variable color for better visualization of individuals.

Cortisol concentrations in milk were highly variable across individuals (Table 1) and, to a lesser extent, across time within individual (intraclass correlation = 0.58, confidence interval: 0.45–0.71, *N* = 108, Figure 1). The concentration of fat in milk was positively associated with cortisol concentrations in milk (estimate ± standard error [SE] = 0.04±0.017, *t* = 2.1, *P* = 0.038, *N* = 108) as was protein concentration (estimate ± SE = 0.3±0.1, *t* = 2.8, *P* = 0.0064, *N* = 108). Milk yield was negatively associated with cortisol in milk (estimate ± SE = −0.02±0.006, *t* = −3.25, *P* = 0.0014, *N* = 108), as higher milk yield diluted the concentration of cortisol. Because cortisol in milk was associated with the caloric content in milk (fat and protein) and the volume of milk (yield), we used the aggregate measure of AME (Hinde 2009; Hinde and Capitanio 2010) as a covariate during AICc model selection to evaluate the independent contributions of cortisol to infant outcomes.

**Table 1:** Mean, standard deviation, and ranges for cortisol concentrations in mother’s milk at early and peak lactation (1 month and 3–4 months postpartum, respectively) and the calculated change (Δ) per day between the 2 time points for rhesus macaques (*N* = 108)

Time point	Concentration (nmol/L)		
	Mean	SD	Range
Early	175.5	86.7	19–444
Peak	172.7	97.3	21–622
Δ per day	−0.06	1.2	−4.5 to 2.8

### Maternal characteristics and cortisol in milk

**Figure 2:**
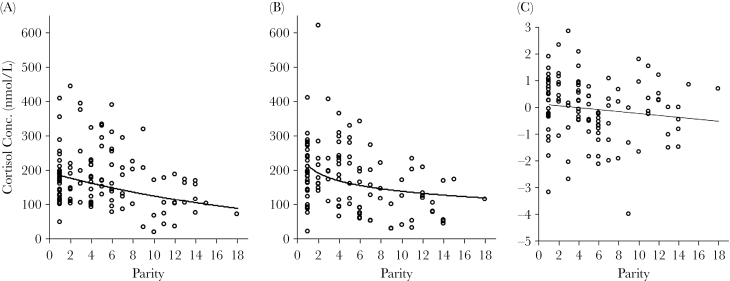
Scatter plot of cortisol concentrations in milk at early (A) and peak (B) lactation as well as the calculated change (Δ) per day between the 2 time points (C) by maternal parity. Fitted regression line in black.

**Figure 3:**
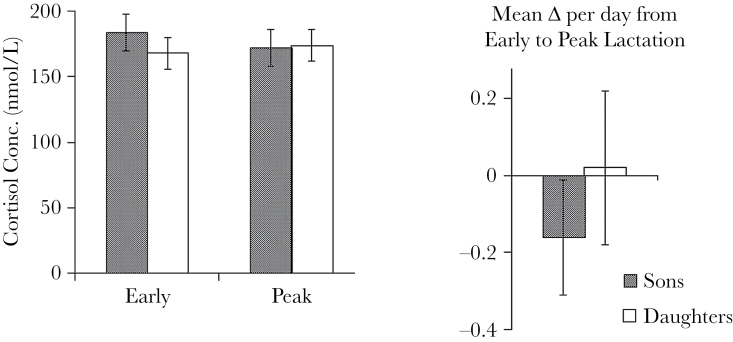
Mean ± SEM of cortisol concentrations in milk of mothers of sons (*N* = 47) and daughters (*N* = 61) at early and peak lactation and the mean calculated change (Δ) per day between the 2 time points.

Parity was the only maternal characteristic that was significantly associated with cortisol concentrations in milk, and the relationship was negative: mothers of lower parity produced higher concentrations of cortisol in milk at both early (full model: adj. *R* ^2^ = 0.18, *F*_4,103_ = 6.8, *P* < 0.0001, *N* = 108, Figure 2A) and peak lactation (full model: adj. *R* ^2^ = 0.26, *F*_5,102_ = 8.5, *P* < 0.0001, *N* = 108, Figure 2B). Similarly, cortisol concentrations in milk decreased to a lesser extent across lactation for lower parity mothers than for higher parity mothers (full model: adj. *R* ^2^ = 0.15, *F*_4,103_ = 5.66, *P* = 0.0004, *N* = 108, Figure 2C). These relationships were nonlinear and AICc model selection revealed slightly steeper slopes across early parities that were attenuated in high parity mothers (Figure 2). Maternal body mass had minimal influence on cortisol concentrations. While accounting for parity, heavier mothers had slightly lower cortisol concentrations in milk at peak lactation only (estimate ± SE = −0.07±0.04, *t* = −1.77, *P* = 0.08, *N* = 108), but this effect was nonsignificant. Maternal mass was not retained in models for cortisol concentrations at early lactation or in changes across lactation. Maternal rank, initially included, was removed as a predictor during AICc model selection (Table 2). Mean concentrations and change of cortisol concentrations in milk did not differ among high-, middle-, and low-ranking mothers (Table 2). Importantly, mothers of sons and mothers of daughters produced milk with the same cortisol concentrations at early and peak lactation and did not differ in the magnitude of change in cortisol concentration across lactation (Figure 3).

**Table 2:** Mean ± SEM of cortisol in milk among high-, middle-, and low-ranking macaque mothers at early and peak lactation (1 month and 3–4 months postpartum, respectively) and the calculated change (Δ) per day between the 2 time points

Rank	*N*	Concentration (nmol/L)		
		Early	Peak	Δ per day
High	39	175±13	169±15	−0.08±0.2
Middle	36	176±15	177±14	0.02±0.2
Low	33	176±16	172±21	−0.12±0.2

### Mother’s milk and infant growth

Mother’s milk was a strong predictor of infant growth (grams per day). Infant growth, controlling for infant body mass at 1 month of age, was greater if cortisol concentration in milk was higher at peak lactation (estimate ± SE = 0.44±0.21, *t* = 2.15, *P* = 0.034) and as AME from the mother increased from early to peak lactation (estimate ± SE = 4.32±1.5, *t* = 2.9, *P* = 0.0044). In this multiple regression model, AICc retained 2 additional parameters although they were not statistically significant. Higher AME at early lactation (estimate ± SE = 0.04±0.02, *t* = 1.73, *P* = 0.09) and lower cortisol concentrations in milk at early lactation (estimate ± SE = −0.34±0.24, *t* = −1.44, *P* = 0.15) predicted greater infant daily growth (full model: adj. *R* ^2^ = 0.24, *F*_5,102_ = 7.73, *P* < 0.0001, *N* = 108).

### Infant confident and nervous temperament predicted by cortisol in milk and other parameters

**Figure 4:**
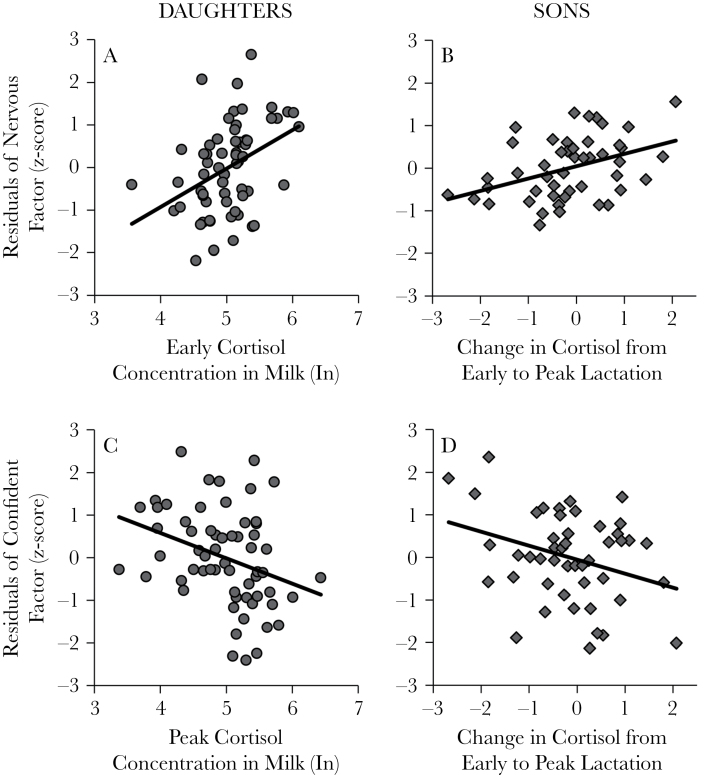
Residualized scatter plots and fitted regression line for milk cortisol parameters and infant temperament factor by infant sex. Nervous temperament of daughters in relation to cortisol concentrations in milk at early lactation (A) and for sons in relation to the change in cortisol concentrations in milk from early to peak lactation (B). Confident temperament of daughters in relation to cortisol concentrations in milk at peak lactation (C) and for sons in relation to the change in cortisol concentrations in milk from early to peak lactation (D).

Milk cortisol parameters predicted infant temperament factors although sons and daughters were differentially sensitive to their mother’s milk cortisol and demonstrated different critical windows of sensitivity. For daughters, Nervous temperament was positively related to absolute concentrations of cortisol in milk during early lactation (estimate ± SE = 1.00±0.29, *t* = 3.48, *P* = 0.001, *N* = 61; Figure 4A), whereas Confident temperament was negatively associated with cortisol concentrations obtained at peak lactation (estimate ± SE = −0.67±0.24, *t* = −2.8, *P* = 0.007, *N* = 61; Figure 4C). For sons, the direction and magnitude of the change in cortisol from early to peak lactation, and not absolute concentrations per se, predicted temperament. Specifically, increases in cortisol concentration predicted higher Nervous (estimate ± SE = 0.36±0.11, *t* = 3.27, *P* = 0.002, *N* = 47) and lower Confident (estimate ± SE = −0.44±0.17, *t* = −2.42, *P* = 0.02, *N* = 47) temperament at BBA for sons (Table 3).

**Table 3:** Maternal, infant, and milk characteristics associated with Nervous (A) and Confident (B) temperament for daughters and sons in rhesus macaques

Predictors	Daughters (*N* = 61)			Sons (*N* = 47)		
	Estimate ± SE	*t*	*P* > |*t*|	Estimate ± SE	*t*	*P* > |*t*|
(A) Outcome: Nervous temperament factor (*z*-score)						
Intercept	−5.06±1.47	−3.45	0.0011	0.42±0.45	0.93	0.356
Early CORT (ln)	1.00±0.29	3.48	0.001	—	—	—
Peak CORT (ln)	—	—	—	—	—	—
Change CORT	0.2±0.11	1.89	0.065	0.36±0.11	3.27	0.002
Early AME	—	—	—	—	—	—
Peak AME	—	—	—	—	—	—
Change AME	—	—	—	4.02±1.4	2.86	0.007
Maternal rank (H&M < L)	−0.27±0.13	−2.00	0.05	—	—	—
Maternal primiparity	—	—	—	−0.91±0.22	−4.23	0.0001
Infant growth	—	—	—	−0.32±0.09	−3.66	0.007
	Model adj. *R* ^2^ = 0.19, *F* = 5.73, *P*-value = 0.0017			Model adj. *R* ^2^ = 0.41, *F* = 8.87, *P*-value = 0.0001		
When cortisol in milk parameter(s) are removed from models						
	Model adj. *R* ^2^ = 0.044, *F* = 3.76, *P*-value = 0.057			Model adj. *R* ^2^ = 0.28, *F* = 6.76, *P*-value = 0.0008		
(B) Outcome: Confident temperament factor (*z*-score)						
Intercept	4.09±1.27	3.22	0.0021	−0.44±0.69	−0.64	0.5281
Early CORT (ln)	—	—	—	—	—	—
Peak CORT (ln)	−0.67±0.24	−2.80	0.007	—	—	—
Change CORT	—	—	—	−0.44±0.17	−2.42	0.02
Early AME	—	—	—	—	—	—
Peak AME	—	—	—	—	—	—
Change AME	—	—	—	−4.58±2.15	−2.13	0.039
Maternal rank	—	—	—	—	—	—
Maternal primiparity	0.64±0.31	2.08	0.04	0.76±0.33	2.32	0.025
Infant growth	—	—	—	0.32±0.14	2.38	0.022
	Model adj. *R* ^2^ = 0.11, *F* = 4.69, *P*-value = 0.013			Model adj. *R* ^2^ = 0.19, *F* = 3.62, *P*-value = 0.013		
When cortisol in milk parameter(s) are removed from models						
	Model adj. *R* ^2^ = 0.007, *F* = 1.40, *P*-value = 0.24			Model adj. *R* ^2^ = 0.09, *F* = 2.58, *P*-value = 0.07		

CORT, cortisol; Predictors listed were initially considered for inclusion in models. Predictors denoted with em dashes in table were not retained during forward and backward AICc model selection. Final model adjusted *R* ^2^, *F*-value, and *P*-values provided for both full model and after removing cortisol predictor from the model.

Other parameters of maternal condition, milk synthesis, and infant condition also predicted infant temperament. However, the covariates retained by AICc model selection to predict temperament generally differed between sons and daughters. “Nervous” factor score for daughters was additionally sensitive to maternal rank. Daughters of low-ranking mothers were significantly more “Nervous” than were the daughters of high- and middle-ranking mothers (estimate ± SE = −0.27±0.13, *t* = −2.00, *P* = 0.05, *N* = 61, Table 3). Growth rate in sons was predictive of temperament; better growth predicted lower “Nervous” (estimate ± SE = −0.32±0.09, *t* = −3.66, *P* = 0.007, *N* = 47, Table 3) and higher “Confident” (estimate ± SE = 0.32±0.14, *t* = 2.38, *P* = 0.022, *N* = 47, Table 3) factor scores. Primiparity predicted higher Confident factor score in both sons and daughters and lower Nervousness factor score in sons. Lastly, stable AME from early to peak lactation predicted lower Nervous and higher Confident factor scores for sons (Table 3).

Collectively, the above parameters explained ~19% and ~11%, respectively, of the variance in daughters’ Nervous and Confident factor scores and ~41% and ~19% of the variance in sons, respectively (model adj. *R* ^2^ provided in Table 3). Exclusion of cortisol in milk parameters dramatically reduced the amount of variance explained by the multiple regression models (Table 3). Given that Nervous and Confidence factor *z*-scores were negatively correlated, we conducted post hoc analyses including one as a predictive parameter for the other. For Nervous factor, all previously associated milk, maternal, and infant characteristics were retained in models as in Table 3A, strengthening model fit (daughter model: *F* = 8.5, *P* < 0.0001, adj. *R* ^2^ = 0.27; son model: *F* = 13.3, *P* < 0.0001, adj. *R* ^2^ = 0.57). However, milk, maternal, and infant characteristics were not retained by AICc model selection in models for Confident factor *z*-score when Nervous factor *z*-score was included as a covariate.

## DISCUSSION

Glucocorticoid concentrations in mother’s milk, independent of AME, were strongly associated with infant temperament in both sons and daughters. Generally, exposure to maternal-origin glucocorticoids via milk was associated with higher Nervous and lower Confident temperament factor scores. During the 25-h BBA, high Nervous animals retreat from others and outside disturbances, are hesitant to venture into new situations, and their fearfulness and timidity causes them to be easily alarmed (Golub et al. 2009). Previously, the BBA program has revealed that when confronted by a “human intruder,” high Nervous animals have higher frequencies of fear grimaces, threats, and barks and spend less time near the intruder (Capitanio et al. 2011). In contrast, high Confident animals are inquisitive, more readily explore new situations and investigate novel situations and objects, are energetic, engaging in more locomotor and object play, and behave in a fearless, bold, and daring manner (Golub et al. 2009).

The factors of Nervous and Confident are consistent, though not identical, with the bold–shy construct that is common in behavioral ecology and animal behavior (Réale et al. 2007; Smith and Blumstein 2008). However, depending on social and ecological conditions—competition for resources and mates, predator density, infectious disease, and the personalities of other individuals—fitness correlates of nervousness and confidence are likely to be highly variable (reviewed in Hinde 2013). Across taxa, boldness is associated with higher reproductive success to a greater extent in males and among captive animals but is associated with reduced survival regardless of ecology (Smith and Blumstein 2008). That meta-analysis, however, primarily used results from studies of subjects assessed in adulthood (Smith and Blumstein 2008), after individuals had successfully survived early development when most mortality occurs. Mother’s milk, an obligate environmental input for developing mammals, may necessarily influence offspring behavioral reaction norms by providing the calories that fuel, and the hormones that influence, behavior in the context of the adaptively relevant environment—the mother.

Our results further indicate sex differences in the windows of sensitivity and the magnitude of sensitivity to maternal-origin glucocorticoids. Daughters were particularly sensitive to absolute concentrations of cortisol in mother’s milk at early and peak lactation: higher cortisol in milk at early lactation was predictive of greater Nervous temperament scores and higher cortisol at peak lactation was associated with lower Confident temperament scores. Daughters were particularly sensitive to cortisol ingested in milk, as few other parameters predicted temperament and explained relatively little of the variance compared with milk cortisol predictors. Sons, in contrast, were more Nervous and less Confident if cortisol concentration increased between early and peak lactation. Sons were particularly sensitive to dynamic signals—specifically changes in milk and their own growth across time—with identical parameters retained in models predicting Nervous and Confident outcomes. Notably these temperament factors, while correlated, had only the single trait adjective “confident” in common. Post hoc analyses in which Confident and Nervous factor scores were included among the predictors for the other in turn revealed that milk cortisol was strongly predictive of infant Nervous temperament even when controlling for Confident temperament factor (but not vice versa), suggesting that the association between milk cortisol and Confident temperament appeared to be mediated through the association with underlying attributes shared between Nervous and Confident factors.

Our results add to the growing literature showing that exposure to maternal glucocorticoids importantly contributes to phenotypic development of offspring (Love et al. 2013). To date, most research has addressed exposure prenatally or prehatching in mammals, birds, fish, and reptiles (reviewed in Coe and Lubach 2000; Seckl 2004; Murphy et al. 2006; Singh et al. 2012; Nyberg 2013; *Larus michahellis*, Rubolini et al. 2005; *Gasterosteus aculeatus*, Giesing et al. 2011; *Lacerta vivipara*, Vercken et al. 2007; *Niveoscincus ocellatus*, Cadby et al. 2010). Maternal glucocorticoids can exert sex-differentiated effects: exposure to elevated glucocorticoids reduced growth particularly in sons (*Homo sapiens*, Thayer et al. 2012; *Ctenophorus fordi*, Uller et al. 2009). However, little is known about the ingestion of maternal-origin glucocorticoids via milk. Our results are partially consistent with previous reports in rodents, primates, and humans by revealing an association between glucocorticoids in milk and behavioral phenotype and sex differences in sensitivity. However, unlike for rodents (reviewed in Macri et al. 2011 and Catalani et al. 2011), glucocorticoids in macaque milk are associated with greater nervousness and lower confidence, more reminiscent of the recent study in humans (Grey et al. 2013). Importantly, our results diverge from our previous finding that cortisol in milk was positively associated with Confident temperament in sons (Sullivan et al. 2011, in which AME was not considered) and our earlier result that absolute AME was positively associated with Confident temperament in infants (Hinde and Capitanio 2010, in which cortisol was not considered). Here, we incorporated dynamic measures of mother’s milk, capturing time points, and changes between them, which were not explored in the previous studies. The substantially larger sample size additionally allowed us to consider more maternal and infant covariates as well as build statistical models for daughters and sons separately. Most importantly, we were able to simultaneously incorporate milk nutrition into our study of milk hormones. For sons especially, the dynamic changes in these parameters, which were not assessed previously, were most predictive of behavioral phenotype.

By simultaneously considering AME, cortisol in milk, and infant growth, we gain some insights into trade-offs for allocating milk energy between growth and behavioral activity and contribute a developmental perspective to the expanding literature on the relationship between metabolism and behavioral phenotype (Careau et al. 2008; Biro and Stamps 2010; Houston 2010; Burton et al. 2011) and behavioral reaction norms (Dingmanse et al. 2010). If more nervous, less confident behavioral phenotypes have reduced behavioral activity budgets, this may facilitate the allocation of milk energy preferentially to growth, particularly for sons. Consistent with this interpretation was our finding that the dynamic increase in AME from early to peak lactation was more predictive of infant growth than was the absolute value of AME. This suggests that when more milk calories become available, growth is prioritized, but at least in sons, this is also associated with being more nervous and less confident. Higher cortisol at peak lactation was also associated with better daily growth in our study, similar to how glucocorticoids can accelerate offspring growth in squirrels (*T. hudsonicus*, Dantzer et al. 2013). Taken together, these findings suggest that in rhesus, maternal-origin cortisol influences trade-offs between growth and behavior, prioritizing growth by diverting energetic expenditure away from behavioral activity by programming a less exploratory, playful, and bold, but more nervous, fearful, and timid infant. Observations of infant behavioral phenotype within the social group, separate from BBA, will be important to clarify how temperament translates into behavioral activity budgets in a more naturalistic context.

This hormonal signature in milk that seemingly signaled “be fearful, prioritize growth” was produced by those mothers confronting crucial life-history trade-offs. Lower parity mothers produced higher cortisol concentrations in milk and showed less of a decrease in cortisol concentration across lactation. Further, controlling for parity, mothers of lower body mass also had slightly higher concentrations of milk cortisol at peak lactation, though not significantly so. Young mothers and poor condition mothers face the greatest challenges for sustaining lactation and produce lower AME (Hinde 2009; Hinde et al. 2009). Young macaque mothers are typically still growing during the early years of their reproductive career (Lipkin et al. 2001; Cerroni et al. 2003; Hinde et al. 2009) and are rearing infants proportionally larger, relative to maternal mass, than are more reproductively experienced females (Hinde 2009). Fundamentally, young macaque mothers have more allocation demands, with fewer resources, to rear a relatively more costly infant. Moreover, young mothers have the greatest residual reproductive value (Williams 1966) and face substantial trade-offs between current and future reproduction (Clutton-Brock 1991; Stearns 1992). Depending on maternal baseline condition and availability of resources, mothers can become depleted during lactation, requiring recovery before subsequent reproduction (*H. sapiens*, Prentice 1980; Shell-Duncan and Yung 2004; *Pan troglodytes*, Thompson et al. 2012; *Papio hamadryas anubis*, Rosetta et al. 2011; reviewed in Robinson 1986). Mothers with lower milk production and reduced somatic resources, due to poor condition or low parity, may be “programming,” through cortisol signaling, behaviorally cautious offspring that prioritize allocation of milk energy to growth but are otherwise energetically less costly. Indeed, experimentally elevated exposure to glucocorticoids via the egg reduced chick begging displays in yellow-legged gulls (*L. michahellis*, Rubolini et al. 2005). In the absence of experimental manipulations, however, the causal role of milk glucocorticoids in shaping these trade-offs in infant macaques remains speculative.

A trade-off that generates a high Nervous, low Confident behavioral phenotype, however, is not without potential long-term fitness costs. In our population of rhesus macaques, infants characterized by a nervous temperament have compromised glucocorticoid-mediated immune function (Capitanio et al. 2011) and adult males characterized by low confidence had altered hypothalamic-pituitary-adrenal axis regulation (Capitanio et al. 2004). For long-lived mammals, maternal inputs may be less predictive of the environment that offspring will encounter in the future. However, maternal care and physiological investment during development is highly predictive of the mother’s capacity to sustain investment for the many months of lactation. As such, offspring can be expected to calibrate their development as a function of the resources available from the mother (Wells 2010, 2014). This early canalization may not produce an adult optimized for maximal reproductive success, but rather a phenotype that is more likely to survive infancy/juvenility, a necessary prerequisite for reproductive success (Kuzawa and Quinn 2009). For example, we can predict that high Nervous, low Confident animals are more hesitant to socially and nonsocially play, energetically expensive activities that may strain the energy available from mother’s milk. As play behavior is hypothesized to enhance behavioral flexibility as well as neural and cognitive development (Fairbanks 2000; Graham and Burghardt 2010; Montgomery 2014), such offspring may experience some deficits in juvenility and adulthood but may be more likely to survive infancy. Constituents and dynamics of milk may reflect, in part, adaptations in mothers operating to program a “cheaper” infant (Love and Williams 2008; Reddon 2012) or adaptations in infants operating to optimally allocate resources transferred from the mother (Wells 2014). More likely, adaptations are operating in both mothers and young, and they may not always be in harmony (Trivers 1974).

Our understanding of the mechanistic physiology associated with glucocorticoids in milk, while limited, can inform the above ultimate interpretations. Glucocorticoids are locally regulated within the mammary gland (Akers 2002; Neville et al. 2002), are critical for lactogenesis (Neville et al. 2002), and prevent apoptosis of mammary gland cells (Berg et al. 2002). Although lower parity mothers produce milk with higher concentrations of cortisol across lactation, this may be due to mammary gland biology and not necessarily the psychological stress of motherhood for inexperienced females. The capacity to synthesize milk increases across parities due to the cumulative effects of sequential lactations on mammary gland architecture (*Halichoerus grypus*, Lang et al. 2012; *B. taurus*, Anderson and Sheffield 1983; Miller et al. 2006). Higher glucocorticoid activity in the mammary gland may, therefore, be compensatory or partially protective of the proportionally fewer cells in the mammary gland and pass into milk as a byproduct of mammary gland physiology. Moreover, cortisol concentrations in milk were lower at each additional parity, for the first 9 parities, further evidence that this effect is not specific to particularly “stressed” or suboptimal infant care of inexperienced mothers. Although maternal behavior is likely influencing behavioral phenotype of the infant independent of the physiological transfer of milk (Champagne 2014; Dettmer et al. 2014), inferences from breastfeeding and formula-feeding mothers suggest that glucocorticoids ingested via milk are more predictive of infant behavioral phenotype than are maternal circulating glucocorticoids (Glynn et al. 2007).

Hormones in mother’s milk, including glucocorticoids, are likely to pass with appreciable bioactivity through the infant’s gastrointestinal tract due in part to its high permeability, especially in the neonate (Peaker and Neville 1991; Neu 2007; Neville et al. 2012). Among rodents, expression of glucocorticoid receptors in the intestinal tract is highest during infancy but declines to adult levels after weaning, suggesting a specific role for binding glucocorticoids ingested via mother’s milk (Pácha 2000). Ingested glucocorticoids are implicated specifically in the maturation of the gastrointestinal tract in the neonate (Henning 1986; Sheard and Walker 1988). Numerous bioactives in milk such as leptin, adiponectin, relaxin, tumor necrosis factor, and others have been linked to infant developmental trajectories (Bartol and Bagnell 2012; Power and Schulkin 2013; Liu et al. 2014). Glucocorticoids, given their diverse roles in metabolism, behavior, and immune function (Hinde 2013; Nyberg 2013), have the potential to exert substantial effects on offspring phenotype.

In many taxa, mothers seemingly synthesize milk tailored to the sex of their young (*H. sapiens*, Powe et al. 2010; Fujita et al. 2012; *M. mulatta*, Hinde 2007, 2009; Hinde et al. 2013; *Myodes glareolus*, Koskela et al. 2009; *C. elaphus hispanicus*, Landete-Castillejos et al. 2005; *Macropus eugenii*, Robert and Braun 2012; *B. taurus*, Hinde et al. 2014). However, in the present study, mothers of sons and mothers of daughters produced milk with the same cortisol concentrations at early and peak lactation and did not differ in the magnitude of change in cortisol concentration across lactation. Similarly, Grey et al. (2013) reported no differences in glucocorticoid concentration in milk for sons and daughters. These findings effectively highlight that the milk a mother synthesizes is only one half of a complex dynamic between mother and infant. Emerging data, including the results presented here, indicate that there are likely sex-specific mechanisms within infants that influence how bioactive constituents in milk are assimilated and utilized (Badyaev 2002; Hinde 2009; Brummelte et al. 2010). In this way, infants must be viewed as active agents in their own development (Fairbanks and Hinde 2013). Where the developmental priorities of males and females diverge, we can predict that sex-specific physiological and behavioral mechanisms have been favored by natural selection to facilitate sex-specific ontogeny.

The precise sex-specific mechanisms underlying the effects reported here in rhesus monkeys are not yet known. We expect that underlying differences in the distribution and density of glucocorticoid receptors in central tissues (as found in rodents: Catalani et al. 2011) and in peripheral tissues, particularly the gastrointestinal tract, are key mediating mechanisms. Importantly, among rhesus macaques, sex-differentiated neurodevelopment has been described for neural regions that underlie memory and emotion processing (Knickmeyer et al. 2010; Raper et al. 2013), areas implicated in anxiety and nervousness (Kim et al. 2011; Haley et al. 2012). Our results indicate that absolute cortisol concentrations were associated with the temperament of daughters but that changing cortisol across time was associated with the temperament of sons. However, this difference may be in part attributable to our sampling regime at only 2, albeit important, time points in behavioral and neurobiological development, during infancy. There could very well be dynamic signals influencing daughters and static time points for sons not captured in the present study, and future research should integrate repeated measures of neurobiological and gastrointestinal development.

Several additional factors predicted infant temperament. Notably, sons of primiparous mothers were characterized as less nervous and more confident than were the sons of multiparous mothers. Daughters of primiparous mothers were characterized as more confident. This is seemingly in conflict with our interpretation that low parity mothers with fewer resources produce more nervous, less confident offspring. However, besides their reduced somatic resources, there is 1 additional feature of primiparous mothers that may be relevant—the lack of previous offspring. The presence of siblings is likely to influence the development of behavioral phenotype (Hudson et al. 2011), especially in taxa in which older siblings are appreciably larger, more developed, and remain in close association with the mother and younger siblings. Although serving as a potential playmate (Pereira and Preisser 1998; Petrů et al. 2009), older siblings may present a risk and effectively be a deterrent from active exploration. We also found that daughters of low-ranked mothers were characterized as more nervous. This is consistent with the natural history of cercopithecines in which daughters inherit social rank from their mothers (Berman 1980b; Chapais 1988; Pereira 1992). Future studies of the development of behavioral phenotype should combine mother’s milk, temperament assessment, infant behavioral activity, and social interactions, especially with siblings, within the social group. Indeed, social experiences beyond the scope of the present study are expected to influence behavioral phenotype (Sachser et al. 2013), but those experienced during infancy are fueled and framed by mother’s milk.

## CONCLUSIONS

The maternal environment, physiological during fetal life and behavioral during infancy, has well-established influences on behavioral development. But an aspect of that maternal environment for mammals remains physiological postnatally as well: the nutrition and hormonal signaling provided through the milk she synthesizes during lactation. The transfer of that milk is behaviorally mediated through nursing dynamics negotiated between mother and infant. In this way, milk is at the intersection of maternal physiology and behavioral care. Just as individuals vary in their “mothering style,” there is substantial variation in milk composition and yield across individuals and across time within individual. Mother’s milk may inform trade-offs and transitions within a hierarchy of priorities during early life: survival, growth, and behavioral activity. The influence of mother’s milk on behavioral phenotype may be especially important among taxa in which mothers are continuously in proximity to their young, and social behavior contributes to fitness outcomes. Previous research has demonstrated that infant baboons suspend growth in favor of maintenance when mother’s milk synthesis is experimentally reduced (Roberts et al. 1985). Here, we show evidence that infant growth may be prioritized over the development of an exploratory, playful, confident temperament and that cortisol ingested via mother’s milk seemingly plays a role in orchestrating this trade-off. The critical windows for these maternal effects were different between sons and daughters, likely reflecting sex-differentiated developmental trajectories and sensitivities. Importantly, these sex differences did not reflect differential cortisol signaling for sons and daughters. The previous studies of cortisol in milk have been presented through the lens of biopsychology; however, by expanding the perspective to incorporate behavioral ecology and life-history theory, we have a better understanding of the natural variation in milk synthesis and the integrated consequences for infant development.

## FUNDING

This research was supported by the National Science Foundation (BCS-0921978 and BCS-0525025 to K.H.), the National Center for Research Resources at the National Institutes of Health (R24RR019970 to J.P.C., P51RR000169 to CNPRC), currently supported by the Office of Research Infrastructure Programs/OD at the National Institutes of Health (R24OD010962 to J.P.C., P51OD011107 to CNPRC).
